# Lipodystrophies in Clinical Practice: A Case Series From a Local Health Unit in Portugal

**DOI:** 10.7759/cureus.103758

**Published:** 2026-02-17

**Authors:** Renata Barbosa, Ana T Pinheiro, Teresa Borges, Ermelinda S Silva, Jorge Diogo Silva, Ana Rita Soares, Rui Carvalho, Liliana Fonseca

**Affiliations:** 1 Division of Endocrinology, Centro Hospitalar e Universitário de Santo António, Unidade Local de Saúde de Santo António, Porto, PRT; 2 Pediatric Endocrinology Unit, Department of Pediatrics, Centro Materno Infantil do Norte, Unidade Local de Saúde de Santo António, Porto, PRT; 3 Pediatric Gastroenterology Unit, Department of Pediatrics, Centro Materno Infantil do Norte, Unidade Local de Saúde de Santo António, Porto, PRT; 4 Medical Genetics Centre Dr. Jacinto Magalhães, Unidade Local de Saúde de Santo António, Porto, PRT; 5 Unit for Multidisciplinary Research in Biomedicine (UMIB), School of Medicine and Biomedical Sciences (ICBAS) University of Porto, Porto, PRT; 6 Laboratory for Integrative and Translational Research in Population Health, ITR - Laboratory for Integrative and Translational Research in Population Health, Porto, PRT; 7 Genetyca-ICM, Atrys, Porto, PRT; 8 Life and Health Sciences Research Institute (ICVS), School of Medicine, University of Minho, Braga, PRT; 9 ICVS/3B’s, PT Government Associate Laboratory, Braga, PRT

**Keywords:** adipogenesis, adipose tissue, hypertriglyceridemia, insulin resistance, lipodystrophy

## Abstract

Background

Lipodystrophies are rare disorders characterized by loss of adipose tissue, leading to severe metabolic and multisystem complications. Data on real-world management remain limited, particularly in Portugal.

Objectives

The objective of this study is to describe the clinical, metabolic, genetic, and therapeutic characteristics of patients with confirmed or suspected lipodystrophy followed at a Portuguese Endocrinology Outpatient Clinic.

Methods

We conducted a retrospective observational study including 21 patients with clinical suspicion or diagnosis of lipodystrophy. Demographic, clinical, laboratory, imaging, and genetic data were collected.

Results

The cohort was predominantly female (90.5%) with a median age at diagnosis of 49 years. Sixteen patients (76.2%) had familial partial lipodystrophy (FPLD), two (9.5%) had congenital generalized lipodystrophy, two (9.5%) had acquired generalized lipodystrophy, and one presented a complex syndromic form. Diabetes mellitus was present in 71.4% of patients and hypertriglyceridemia in 52.4%. Metabolic liver disease occurred in both generalized and partial forms. Autoimmune disorders affected 31.6% of patients, and cardiac involvement was observed in 23.8%. Genetic testing identified pathogenic or likely pathogenic variants in *BSCL2* and *PPARG* in three patients, while most FPLD cases remained genetically unexplained. Metreleptin therapy in three patients with generalized lipodystrophy improved glycemic control, triglycerides, liver enzymes, and proteinuria. Dual-energy X-ray absorptiometry imaging supported the phenotypic characterization of adipose tissue loss.

Conclusions

Detailed physical examination, genetic testing, imaging, and early therapeutic interventions are critical for management. These findings align with European registry data and highlight the need for increased awareness and systematic evaluation in real-world clinical practice.

## Introduction

Lipodystrophies are rare disorders characterized by a deficit of adipose tissue without evidence of nutritional deprivation or a catabolic state [1,2]. Depending on the etiology, they are classified as either genetic or acquired and can also be categorized as generalized, partial, or localized, according to the distribution of adipose tissue loss [1,2]. Congenital generalized lipodystrophy (CGL) comprises four main subtypes, caused by pathogenic variants in the genes *AGPAT2* (CGL1), *BSCL2* (CGL2), *CAV1* (CGL3), and *PTRF* (CGL4). Familial partial lipodystrophy (FPLD) encompasses at least six subtypes, including FPLD1 (Köbberling), *LMNA*-related FPLD (Dunnigan type), *PPARG*-related FPLD, and *PLIN1*-, *CIDEC*-, or *LIPE*-associated forms, which display variable patterns of regional fat loss and metabolic severity [2].

The prevalence of lipodystrophies remains challenging to establish, as most data come from case reports and small clinical series. Overall, global prevalence is estimated at approximately 1.3-4.7 cases per million inhabitants [3]. Congenital forms are exceptionally rare; CGL occurs in about 1 per 10 million individuals, while FPLD affects fewer than 1 per 100,000. Population-based estimates for acquired lipodystrophy suggest a prevalence of 0.2-1 per million for generalized and 1.7-2.8 per million for partial forms [4-6].

There is significant phenotypic variability among the different subtypes of lipodystrophies [1]. On physical examination, patients may present with prominent musculature, phlebomegaly, acanthosis nigricans, and abdominal distension [2,7].

Lipodystrophies are associated with multiple comorbidities, including hypertriglyceridemia, insulin resistance, diabetes, metabolic dysfunction-associated steatohepatitis (MASH), metabolic dysfunction-associated steatotic liver disease (MASLD), liver cirrhosis, chronic kidney disease, and cardiomyopathy [1,2,7].

A key pathophysiological mechanism across lipodystrophy subtypes is leptin deficiency, which plays a critical role in the development of severe insulin resistance, hypertriglyceridemia, ectopic lipid accumulation, and metabolic liver disease [1,2,7].

Diagnosis of lipodystrophies is often challenging. Although no validated diagnostic criteria currently exist, diagnosis is primarily clinical and relies on the identification of generalized or partial loss of subcutaneous adipose tissue, in association with severe metabolic abnormalities such as insulin resistance, hypertriglyceridemia, and metabolic liver disease, and exclusion of secondary causes. Imaging techniques and genetic testing may support the diagnosis but are not mandatory [1,2]. In certain subtypes, the co-occurrence of autoimmune diseases may aid in diagnosis.

In addition to implementing healthy lifestyle measures and treating the associated metabolic complications, metreleptin therapy can be used in cases of CGL or acquired generalized lipodystrophy (AGL) [1,2,8].

Despite growing awareness and the development of international registries, local real-world data remain limited. This study aims to present the experience of a Portuguese Local Health Unit in diagnosing and managing patients with diagnosis or clinical suspicion of lipodystrophy followed at the Endocrinology Outpatient Clinic.

## Materials and methods

This was a retrospective, observational study conducted at the Endocrinology Outpatient Clinic of the Unidade Local de Saúde de Santo António, Porto, Portugal. Case identification and electronic medical record review were performed between September and December 2025.

Patients were eligible for inclusion if they had a documented clinical suspicion or diagnosis of lipodystrophy based on electronic medical records, defined by the presence of generalized or partial loss of subcutaneous adipose tissue, associated metabolic abnormalities suggestive of adipose tissue deficiency, and exclusion of secondary causes of adipose tissue loss. The duration of follow-up was not an inclusion criterion and varied between patients, reflecting the real-world nature of this retrospective case series. Exclusion criteria were individuals with adipose tissue redistribution secondary to HIV infection/antiretroviral therapy, total body irradiation or hematopoietic stem cell transplantation, localized forms of lipodystrophy, or insufficient clinical data to allow comprehensive analysis.

Due to the absence of validated diagnostic criteria for lipodystrophies, clinical suspicion and diagnosis were based on a combination of indicators, including generalized or partial loss of subcutaneous adipose tissue, consistent with features described in the literature, metabolic abnormalities suggestive of adipose tissue deficiency, and exclusion of secondary causes [1]. Supportive diagnostic assessments included leptin levels, dual-energy X-ray absorptiometry (DXA)-derived body fat distribution, genetic testing, and immunological markers when available. Complex lipodystrophic syndromes were considered in cases of lipoatrophy accompanied by multisystem involvement, dysmorphic features, or premature aging, reflecting broader genetic or inflammatory disorders [1].

**Figure 1:**
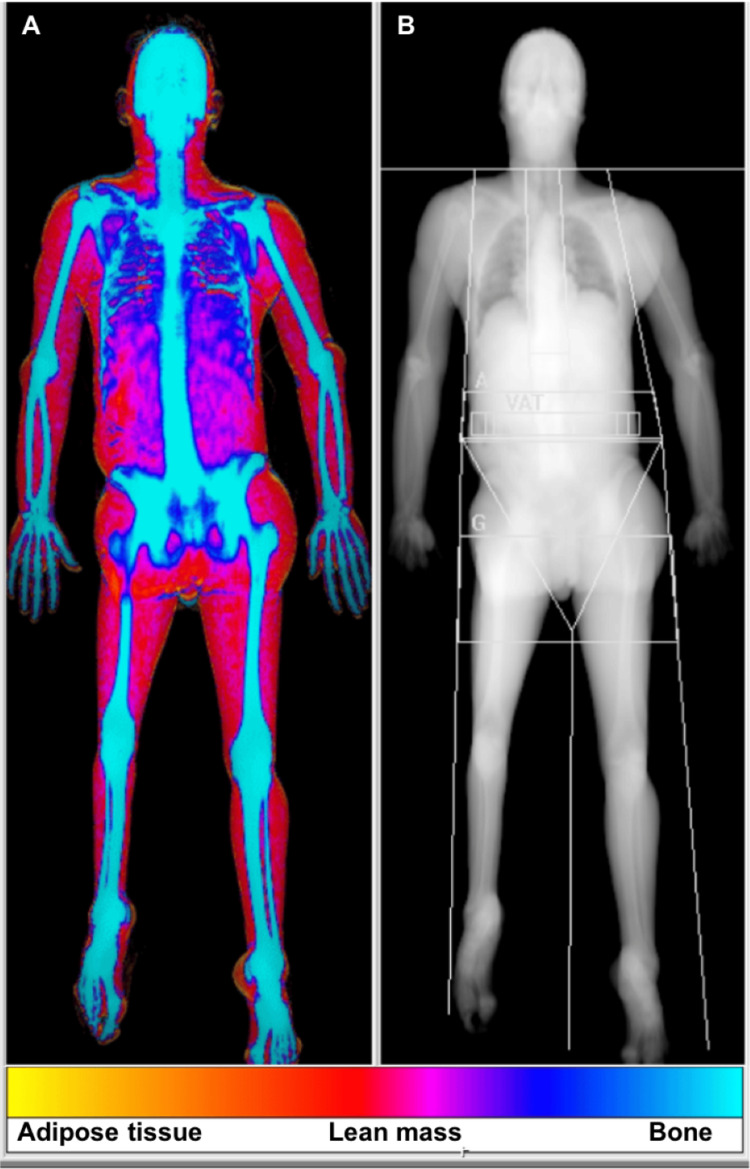
Whole-body dual-energy X-ray absorptiometry scan illustrating body composition analysis. The color-coded image (A) differentiates adipose tissue, lean mass, and bone, while the grayscale image (B) represents the corresponding anatomical distribution.

For each patient, demographic characteristics (age and sex), lipodystrophy classification, clinical variables, and laboratory data were extracted from electronic medical records. Clinical data included anthropometric measurements, medication use, and findings from the first and most recent consultations. Laboratory parameters included glucose metabolism, leptin, lipid profile, liver enzymes, and renal markers, including urinary albumin excretion. Complications affecting metabolic, hepatic, renal, or cardiovascular systems were systematically assessed. MASLD, MASH, and cirrhosis were diagnosed based on a combination of biochemical abnormalities (persistent elevation of liver enzymes), imaging findings (abdominal ultrasound, computed tomography, or magnetic resonance imaging demonstrating hepatic steatosis), and/or liver histology when available [2]. Body composition assessed by DXA and genetic testing results were also reviewed. A representative DXA image illustrating generalized adipose tissue loss is shown in Figure 1.

Statistical analysis was performed using IBM SPSS Statistics for Windows, Version 29 (Released 2023; IBM Corp., Armonk, New York, United States). Categorical variables are presented as numbers and percentages. For continuous variables, normal distribution was tested through kurtosis and skewness analysis. Results are presented as mean values ± standard deviation and median values (minimum-maximum; interquartile range (IQR)), as appropriate. Continuous variables were analyzed longitudinally by comparing first and last appointments using the Wilcoxon signed-rank test for paired non-parametric data, with results presented as test statistics and corresponding p-values. A p-value < 0.05 was defined as denoting a significant difference. 

The authors confirm that the study followed ethical guidelines according to the Clinical Research and Ethics Committee and the Helsinki Declaration and in accordance with institutional protocols.

## Results

Subtypes and general characteristics

Twenty-one patients with a documented clinical suspicion or diagnosis of lipodystrophy were included. The median duration of clinical follow-up documented in medical records was 61 months (minimum-maximum: 6-339 months). Nineteen patients (90.5%) were female. The median age at diagnosis was 49 years (0-66, IQR 39). Sixteen patients (76.2%) had FPLD, two (9.5%) had CGL, two (9.5%) had AGL, and one case (4.8%) had lipodystrophy associated with a complex lipodystrophic syndrome.

The majority of cases were classified as having a congenital etiology (n=19, 90.5%), including patients with genetically confirmed congenital lipodystrophy as well as one patient with a complex syndromic form with early-onset disease, while the remaining cases corresponded to acquired forms. All patients exhibited signs of fat redistribution.

Detailed demographic and clinical characteristics of the entire population are presented in Table 1. Subsequent analyses were stratified by lipodystrophy subtype (partial or generalized) to highlight subtype-specific clinical and metabolic features, as these subtypes present distinct profiles with different genetic backgrounds and pathophysiological mechanisms, which may influence outcomes and biomarker patterns, allowing identification of features that could be masked in the overall cohort.

**Table 1:** Demographic and clinical characteristics of the total population BMI: Body mass index; MASLD: metabolic dysfunction-associated steatotic liver disease; MASH: metabolic dysfunction-associated steatohepatitis; PCOS: polycystic ovary syndrome. **Values are shown as median (interquartile range (IQR))

Characteristics	n (%) or median (IQR)
Total patients	21 (100)
Age at diagnosis** (years)	49 (38.5)
Female gender n (%)	19 (90.5)
BMI** (kg/m^2^)	26.5 (9.2)
Diabetes mellitus n (%)	15 (71.4)
Diabetes mellitus complications	
Retinopathy, n (%)	4 (26.7)
Kidney disease, n (%)	8 (53.3)
Peripheral neuropathy, n (%)	0 (0)
Ischemic cardiopathy, n (%)	1 (6.7)
Peripheral arterial disease, n (%)	1 (6.7)
Cerebrovascular disease, n (%)	2 (13.3)
Hypertriglyceridemia, n (%)	12 (57.1)
Hypertrophic cardiomyopathy n (%)	3 (14.3)
MASLD	7 (33.3)
MASH	2 (9.5)
Cirrhosis n (%)	3 (14.3)
Pancreatitis n (%)	1 (4.8)
PCOS n (%)	4 (19.0)
Hypertension n (%)	8 (38.1)
Malignancy n (%)	1 (4.8)
Intervertebral disc herniations (%)	4 (19.0)
Autoimmune diseases	
Gastritis n (%)	1 (4.8)
Thyroiditis n (%)	6 (28.6)
Hepatitis n (%)	2 (9.5)
Lupus n (%)	1 (4.8)
Psoriasis (%)	1 (4.8)
Thrombocytopenia (%)	1 (4.8)

Generalized lipodystrophies

Metabolic Comorbidities and Associated Clinical Manifestations

In our cohort, four patients (19.0%) were diagnosed with generalized lipodystrophy, including two cases with confirmed Berardinelli-Seip syndrome and two with Lawrence syndrome. Detailed demographic and clinical characteristics are presented in Table 2, while longitudinal metabolic changes are shown in Table 3. Given the small sample size, statistical comparisons should be interpreted cautiously.

**Table 2:** Demographic and clinical characteristics of population (generalized lipodystrophy) BMI: Body mass index; MASLD: metabolic dysfunction-associated steatotic liver disease; MASH: metabolic dysfunction-associated steatohepatitis; PCOS: polycystic ovary syndrome. **Values are shown as median (minimum–maximum)

Characteristics	n (%) or median (min-max)
Age at diagnosis (years)**	2 (0-4)
Female gender n (%)	3 (75)
BMI (kg/m^2^)**	19.7 (19.6-26.3)
Diabetes mellitus n (%)	4 (100)
Diabetes mellitus complications	
Retinopathy, n (%)	1 (25)
Kidney disease, n (%)	4 (100)
Peripheral neuropathy, n (%)	0 (0)
Ischemic cardiopathy, n (%)	0 (0)
Peripheral arterial disease, n (%)	0 (0)
Cerebrovascular disease, n (%)	0 (0)
Hypertriglyceridemia, n (%)	3 (75.0)
Hypertrophic cardiomyopathy n (%)	2 (50)
MASLD	1 (25)
MASH	1 (25)
Cirrhosis n (%)	2 (50)
Pancreatitis n (%)	1 (25)
PCOS n (%)	2 (50)
Hypertension n (%)	1 (25)
Malignancy n (%)	0 (0)
Intervertebral disc herniations (%)	2 (50)
Autoimmune diseases	
Gastritis n (%)	1 (25)
Thyroiditis n (%)	2 (50)
Hepatitis n (%)	1 (25)
Lupus n (%)	1 (25)

**Table 3:** Differences in metabolic parameters of lipodystrophy syndromes at first and last visits (generalized lipodystrophy) ALT: Alanine aminotransferase; AST: aspartate aminotransferase; BMI: body mass index; GGT: gamma-glutamyl transferase; HbA1c: hemoglobin A1c; HDL-C: high-density lipoprotein cholesterol; LDL-C: low-density lipoprotein cholesterol. Leptin reference ranges vary according to BMI: 0.7–8.3 ng/mL if BMI 18.5–24.9; 1.5–19.3 ng/mL if BMI 25.0–30.0; 4.0–32.0 ng/mL if BMI >30. **Values are shown as median (minimum–maximum). Comparisons between first and last appointments were performed using the Wilcoxon signed-rank test for paired non-parametric data.

	Reference range	First appointment	Last appointment	Test statistic (p-value)
HbA1c** (%)	4.0-6.0	6.1 (5.2-7.1)	6.1 (5.6-7.4)	Z=-0.365 (0.715)
Insulin (uU/mL) **	2.6-24.9	37.6 (35.9-60.1)	38.6 (8.9-67.0)	Z= -0.535 (0.593)
C-peptide (ng/mL) **	1.1-4.4	4.3 (3.8-6.3)	5.4 (4.6-6.9)	Z= -1.604 (0.109)
Total cholesterol (mg/dL) **	0-200	128.5 (103.0-140.0)	106.5 (94.0-158.0)	Z=-0.730 (0.465)
LDL-C (mg/dL) **	0-130	45 (45-63)	56.5 (44-92)	Z=-0.447 (0.655)
HDL-C (mg/dL) **	45-65	21.0 (20.0-37.0)	34.5 (32.0-36.0)	Z=-1.069 (0.285)
Triglycerides (mg/dL) **	35–135	238.0 (93.0-367.0)	106.0 (38.0-166.0)	Z=-1.461 (0.144)
GGT (IU/L) **	6-39	34.0 (14.0-48.0)	21.0 (14.0-50.0)	Z=-1.342 (0.180)
AST (IU/L) **	10-30	40.0 (24.0-98.0)	30.5 (11-41)	Z=-1.604 (0.109)
ALT (IU/L) **	10-36	58.5 (54.0-186)	41.0 (31.0-62.0)	Z=-1.826 (0.070)
Leptin** (ng/mL)	0.7-8.3 if BMI 18.5-24.9	0.15 (0.04-0.9)		--
	1.5-19.3 if BMI 25.0-30.0			
	4.0-32.0 if BMI >30			

Diabetes mellitus was present in all four of these patients, one of whom was treated with insulin (basal-only regimen), two (50.0%) with metformin, three (75.0%) with GLP-1 receptor agonists and three (75.0%) with SGLT2 inhibitors. None were treated with DPP-4 inhibitors or pioglitazone.

Median hemoglobin A1c was 6.1% (5.2-7.1), median insulin was 37.6 uU/mL (35.9-60.1), and C-peptide was 4.3 ng/mL (3.8-6.3).

Hypertriglyceridemia was observed in three cases (75.0%), and one patient had a history of pancreatitis associated with hypertriglyceridemia. Lipid profiles showed median total cholesterol levels of 128.5 mg/dL (103.0-140.0), HDL-cholesterol 21.0 mg/dL (20.0-37.0), LDL-cholesterol 45.0 mg/dL (45-63), and triglycerides of 238 mg/dL (93.0-367.0).

None of these patients were receiving statins or ezetimibe, while two (50%) were on fibrate therapy. Additionally, no patients were treated with bempedoic acid or icosapent ethyl.

MASH was documented in one (25.0%) patient, MASLD was documented in one (25.0%) patient, and two (50.0%) had established cirrhosis. Proteinuria was present in all four of these patients, and three (75.0%) showed cardiac involvement (two patients diagnosed with hypertrophic cardiomyopathy and one with lipidic infiltration documented in cardiac magnetic resonance imaging - MRI). Two (50.0%) patients presented with relevant osteoarticular alterations (namely intervertebral disc herniations).

Two (50%) patients were diagnosed with autoimmune diseases, one with autoimmune gastritis, thyroiditis, and hepatitis, and the other with autoimmune thyroiditis and lupus.

Serum leptin measured in these four cases showed consistently low values (median 0.15 ng/mL; minimum-maximum: 0.04-0.9).

Genetic Testing

All these patients underwent genetic testing, with pathogenic variants identified in two of them, namely *BSCL2 *variants (c.325dup; p.Thr109AsnfsTer5 and c.766-49T>C). *BSCL2* encodes seipin, a protein essential for adipocyte differentiation and lipid droplet formation, confirming the diagnosis of Berardinelli-Seip syndrome [2]. In the other two cases, targeted sequencing of known lipodystrophy genes and extended next-generation sequencing approaches, including whole-exome sequencing, did not reveal pathogenic variants. The absence of a genetic diagnosis, together with the clinical features, prompted further investigation, including anti-perilipin antibody testing, which was positive in both cases [9].

Specific Treatment

Three patients with generalized lipodystrophy were treated with metreleptin, showing clinical benefit and improvement in laboratory parameters, with reduction of hemoglobin A1c, proteinuria, and triglycerides. Liver function tests revealed a marked reduction in one patient. Individual metabolic responses to metreleptin therapy are detailed in Table 4.

**Table 4:** Differences in metabolic parameters before and after metreleptin ALT: Alanine aminotransferase; AST: aspartate aminotransferase; GGT: gamma-glutamyl transferase; HbA1c: Hemoglobin A1c; HDL-C: high-density lipoprotein cholesterol; LDL-C: low-density lipoprotein cholesterol. Albuminuria is expressed in mg/g creatinine (mg/gCr).

	Patient 1		Patient 2		Patient 3	
	Male, age 29		Female, age 21		Female, age 14	
	Berardinelli-Seip Syndrome		Lawrence Syndrome		Berardinelli-Seip Syndrome	
	Current dose 5.80 mg/d		Current dose 3.00 mg/d		Current dose 4.64 mg/d	
	Before metreleptin	After metreleptin	Before metreleptin	After metreleptin	Before metreleptin	After metreleptin
HbA1c (%)	6.6	5.0	6.0	5.6	6.9	5.5
Total cholesterol (mg/dL)	110	88	172	158	124	95
LDL-C (mg/dL)	44	34	78	91	44	50
HDL-C (mg/dL)	23	27	17	33	34	37
Triglycerides (mg/dL)	266	136	383	166	232	38
GGT (IU/L)	61	48	223	25	37	17
AST (IU/L)	27	27	733	41	26	11
ALT (IU/L)	33	28	885	62	44	31
Albuminuria (mg/gCr)	104	25	66	30	64	25

Notably, both female patients receiving metreleptin required oophorectomy; one developed a 6.5 cm complex cyst of the right ovary (histopathology consistent with serous cystadenoma), and the other was found to have a paratubal serous cystadenoma (26 mm in greatest diameter).

Imaging Assessment

All four patients underwent body composition assessment and showed a markedly reduced fat mass, both globally and in the limbs and trunk.

Partial lipodystrophies

Metabolic Comorbidities and Associated Clinical Manifestations

Sixteen patients (76.2%) were considered to have partial lipodystrophy, based on clinical history, physical examination, and characteristic patterns of fat distribution on DXA, with or without supportive genetic findings. Detailed demographic and clinical characteristics, as well as the evolution of metabolic parameters of lipodystrophy syndromes between the first and last visits, are presented in Tables 5, 6, respectively.

**Table 5:** Demographic and clinical characteristics of population (partial lipodystrophy) BMI: Body mass index; MASLD: metabolic dysfunction-associated steatotic liver disease; MASH: metabolic dysfunction-associated steatohepatitis; PCOS: polycystic ovary syndrome. **Values are shown as median (interquartile range (IQR))

Characteristics	n (%) or median (IQR)
Age at diagnosis (years)**	51 (15)
Female gender n (%)	15 (93.8)
BMI (kg/m^2^)**	26.6 (4.7)
Diabetes mellitus n (%)	11 (68.8)
Diabetes mellitus complications	
Retinopathy, n (%)	3 (27.3)
Kidney disease, n (%)	4 (36.4)
Peripheral neuropathy, n (%)	0 (0)
Ischemic cardiopathy, n (%)	1 (9.1)
Peripheral arterial disease, n (%)	1 (9.1)
Cerebrovascular disease, n (%)	2 (18.2)
Hypertriglyceridemia, n (%)	8 (50.0)
Hypertrophic cardiomyopathy n (%)	1 (6.3)
MASLD	6 (37.5)
MASH	1 (6.3)
Cirrhosis n (%)	1 (6.3)
Pancreatitis n (%)	0 (0)
PCOS n (%)	2 (12.5)
Hypertension n (%)	7 (43.8)
Malignancy n (%)	1 (6.3)
Intervertebral disc herniations (%)	2 (12.5)
Autoimmune diseases	
Thyroiditis n (%)	3 (18.9)
Psoriasis (%)	1 (6.3)

**Table 6:** Differences in metabolic parameters of lipodystrophy syndromes at first and last visits (partial lipodystrophy) ALT: Alanine aminotransferase; AST: aspartate aminotransferase; BMI: body mass index; GGT: gamma-glutamyl transferase; HbA1c: hemoglobin A1c; HDL-C: high-density lipoprotein cholesterol; LDL-C: low-density lipoprotein cholesterol. Leptin reference ranges vary according to BMI: 0.7–8.3 ng/mL if BMI 18.5–24.9; 1.5–19.3 ng/mL if BMI 25.0–30.0; 4.0–32.0 ng/mL if BMI >30. **Values are shown as median (minimum–maximum; interquartile range [IQR]). Comparisons between first and last appointments were performed using the Wilcoxon signed-rank test for paired non-parametric data.

	Reference range	First appointment	Last appointment	Test statistic (p-value)
HbA1c** (%)	4.0-6.0	7.1 (5.1-10.6; 4.0)	6.2 (4.5-9.2; 2.4)	Z=-1.512 (0.130)
Insulin (uU/mL) **	2.6-24.9	17.2 (6.5- 79.3; 31.2)	14.0 (7.1-68.5; 42.5)	Z=-0.535 (0.593)
C-peptide (ng/mL) **	1.1-4.4	3.7 (1.9-7.0; 2.7)	4.3 (1.4-5.6; 3.5)	Z=-0.730 (0.465)
Total cholesterol (mg/dL) **	0-200	191.0 (109.0-328.0; 108.0)	137.0 (79.0-222.0; 65.0)	Z=-1.005 (0.315)
LDL-C (mg/dL) **	0-130	107.0 (23.0-163.0; 97.0)	63.0 (26.0-129.0; 60.0)	Z=-0.408 (0.683)
HDL-C (mg/dL) **	45-65	45.0 (12.0-66.0; 14.0)	44.0 (14.0-61.0; 20.0)	Z=-0.597 (0.550)
Triglycerides (mg/dL) **	35–135	210.0 (50.0-2139.0; 228.5)	108 (71.0-341.0; 94.0)	Z=-1.874 (0.061)
GGT (IU/L) **	6-39	30.0 (14.0-61.0; 25.0)	24.5 (11.0-193.0;36.8)	Z=-0.629 (0.530)
AST (IU/L) **	10-30	24.0 (12.0-37.0; 16.8)	22.5 (12.0-70.0; 21.5)	Z=-0.070 (0.944)
ALT (IU/L) **	10-36	22.0 (11.0-56.0; 19.0)	21.5 (11.0-94.0; 9.5)	Z=-0.078 (0.937)
Leptin** (ng/mL)	0.7-8.3 if BMI 18.5-24.9	5.2 (2.6-37.9; 5.5)		--
	1.5-19.3 if BMI 25.0-30.0			
	4.0-32.0 if BMI >30			

Diabetes mellitus was present in 11 (68.8%) patients, seven of whom were treated with insulin (four patients with basal-bolus regimen and other three with basal-only regimen), 12 (75.0%) with metformin, 10 (62.5%) with GLP-1 receptor agonists, 10 (62.5%) with SGLT2 inhibitors, two (12.5%) with DPP-4 inhibitors, and one (6.3%) with pioglitazone.

Median hemoglobin A1c was 7.1% with IQR 4.0; the median C-peptide level was 3.7 ng/mL with IQR 2.7.

Hypertriglyceridemia was observed in eight cases (50.0%) and none of the patients had a history of pancreatitis associated with hypertriglyceridemia. Lipid profiles showed median total cholesterol levels of 191 mg/dL (IQR 108), HDL-cholesterol 45.0 mg/dL (IQR 14.0), LDL-cholesterol 107 mg/dL (IQR 97.0) and triglycerides of 135 mg/dL (IQR 228.5).

Among these patients, one (6.3%) was treated with low-intensity statin therapy, four (25.0%) with moderate-intensity statins, and seven (43.8%) with high-intensity statins, eight (50.0%) were treated with ezetimibe and five (31.3%) were on fibrate therapy. None of the patients were treated with bempedoic acid or icosapent ethyl.

MASH was documented in one patient (6.3%), MASLD was documented in six patients (37.5%), and one (6.3%) had established cirrhosis. Proteinuria was present in four patients (36.4%), and two showed cardiac involvement (one patient diagnosed with hypertrophic cardiomyopathy and one with ischemic heart disease). Three patients presented with relevant osteoarticular alterations (two with intervertebral disc herniations and one with scoliosis).

Lipomas were not reported in any case. Other clinical manifestations included infertility (n=1) and hyperphagia (n=2). Three patients (18.8%) had autoimmune diseases, two diagnosed with autoimmune thyroiditis and one with autoimmune thyroiditis and psoriasis.

Serum leptin was measured in 13 patients from this group, with a median value of 5.2 ng/mL (IQR 5.5).

Genetic Testing

Eleven patients (68.8%) underwent genetic testing. Pathogenic or likely pathogenic variants were identified in two cases: one patient underwent whole-exome sequencing, which revealed a heterozygous likely pathogenic variant in* PPARG* (c.581G>A; p.Arg194Gln), and another patient was investigated using a next-generation sequencing lipodystrophy gene panel, identifying heterozygous variants of uncertain significance (VUS) in *CAV1* (c.473C>T; p.Pro158Leu and c.512G>T; p.Arg171Leu). In the remaining nine cases, genetic testing yielded negative or inconclusive results.

Regarding the molecular function of the detected pathogenic variant, *PPARG* encodes a nuclear receptor that regulates adipogenesis, adipocyte survival, and insulin signaling [3,10].

Imaging Assessment

Seven out of 16 patients (43.8%) underwent DXA to quantify body fat and evaluate regional fat distribution, aiming to characterize the pattern of adipose tissue loss. The median trunk-to-limb fat mass ratio was 1.7 (0.9-3.26, IQR 1.36).

Lipodystrophy associated with a complex syndrome

Some forms of lipodystrophy cannot be classified as purely generalized or partial and instead occur in the context of complex syndromes [1].

In our cohort, we report a case of a 22-year-old woman with global developmental delay and fat loss in the limbs since early childhood presents an atypical lipodystrophy pattern associated with a complex syndrome. She has neurodegenerative brain iron accumulation with congenital ataxia and multiple autoimmune disorders (autoimmune hepatitis, autoimmune thrombocytopenia, autoimmune thyroiditis). Additional findings include kyphoscoliosis, clinical signs of severe insulin resistance and hypertriglyceridemia. Genetic testing included direct sequencing of *AGPAT2* and *LMNA*, with additional deletion/duplication analysis of *LMNA* by MLPA (multiplex ligation-dependent probe amplification) and did not identify pathogenic variants.

## Discussion

Subtype distribution and referral patterns

Regarding the subtypes of lipodystrophies, FPLD was the most frequent (76.2%) in our study. This finding is consistent with the European Lipodystrophy Registry (ECLip) [11], a large multicenter study in which FPLD accounted for 57.4% of cases. The predominance of FPLD in both our series and the broader European cohort is in line with the literature and supports the notion that, excluding HIV-related forms, the most common lipodystrophy syndromes are FPLD, typically inherited in an autosomal dominant manner and caused by variants in genes involved in adipogenesis and lipogenesis [3].

In clinical practice, congenital subtypes typically predominate at referral centers, related to their earlier onset and more pronounced phenotype, whereas acquired forms may be underdiagnosed, particularly when they present as partial lipodystrophy or are associated with autoimmune or inflammatory conditions. Accordingly, the proportion of congenital versus acquired cases in our study may reflect a referral bias to endocrinology, an easier diagnosis in more striking forms, and a possible underdiagnosis of acquired forms [12-14].

In our cohort, women were predominant, representing 90.5% of cases. This female predominance is also consistent with previous European reports, emphasizing how gender influences referral patterns into specialized networks [15,16]. This apparent diagnostic bias may be related both to disease-specific characteristics that are more clinically evident in women and to social factors, including the stigmatization of lipodystrophic phenotypes in women [15]. Therefore, it is fundamental to reinforce the importance of considering potential gender-related differences in the clinical recognition and referral patterns of lipodystrophy.

Metabolic complications

In this case series, diabetes mellitus was highly prevalent, affecting 71.4% of patients across the whole cohort, 100% of patients in the generalized group and 68.8% in the partial lipodystrophy group. Among patients with diabetes, kidney disease was the most common complication (53.3%), followed by retinopathy (26.7%) and cerebrovascular disease (13.3%). These results are consistent with the high metabolic burden described in other populations. For instance, in a Spanish cohort, diabetes was reported in 43.5% of patients, with a substantial burden of complications: kidney disease in 36.0%, and retinopathy and peripheral neuropathy, each affecting 20.0% of patients [14]. A Greek cohort reported diabetes in approximately 65% of patients with FPLD [16]. Similarly, the ECLip study reported an overall prevalence of diabetes of 48.4%, but with substantial variation between subtypes (76.9% in acquired partial lipodystrophy and 8.7% in acquired localized lipodystrophy), highlighting the heterogeneity of metabolic risk across different lipodystrophy forms [11].

The substantial proportion of patients requiring insulin therapy in our study is in line with previous ones showing that severe insulin resistance in lipodystrophy often leads to early and intensive insulin requirements [17]. This reinforces the well-established association between the extent of adipose tissue loss and the severity of glycemic dysregulation [17].

Hypertriglyceridemia is also one of several multisystem metabolic complications of lipodystrophy, arising secondary to adipose tissue deficiency that promotes ectopic lipid accumulation and, consequently, severe insulin resistance. Leptin is also a key regulator of lipid metabolism: its absence, particularly in generalized lipodystrophy, prevents activation of JAK/STAT and AMPK pathways, leading to increased lipid synthesis, ectopic fat deposition, lipotoxicity, and systemic metabolic complications [18]. Mechanisms related to a compromised lipoprotein lipase availability in the context of adipose tissue loss are also involved [19].

Additionally, hypertriglyceridemia increases cardiovascular risk, as chylomicron remnants and other triglyceride-rich lipoprotein particles can penetrate the arterial wall and promote endothelial dysfunction and vascular inflammation. Notably, these remnants are larger than LDL particles and can carry approximately twice the cholesterol load, amplifying their atherogenic potential. Thus, the combination of adipose tissue deficiency, impaired triglyceride clearance, and elevated remnant lipoproteins in lipodystrophy creates a pro-atherogenic environment that markedly increases cardiovascular risk [20].

Our case series exhibited a relevant prevalence of hypertriglyceridemia (52.4%), similar to other cohorts, and one patient with a history of pancreatitis [14-16]. This prevalence reinforces that dyslipidemia is an early and prominent metabolic feature in lipodystrophy, even before the occurrence of severe forms, and highlights the importance of close monitoring to prevent complications. In our retrospective assessment, triglyceride levels showed a clinically relevant decline from the first to the most recent evaluation, although this change did not reach statistical significance.

Moreover, metabolic liver involvement is highly prevalent in lipodystrophies, a pattern also observed in our case series: in generalized forms, MASLD and MASH were each present in 25% of patients, with cirrhosis in two cases (50%); among partial forms, MASLD occurred in 37.5%, MASH in 6.3%, and cirrhosis in one patient (6.3%). These findings are consistent with data from other European cohorts: in a Greek referral center, 56.4% of patients with partial lipodystrophy had MASLD/MASH, and in the ECLip Registry, MASLD and MASH were reported in 38.1% and 12% of individuals, respectively, with cirrhosis present in 1.7% [11,16]. Together, these observations reinforce that hepatic steatosis and its inflammatory progression represent major and common complications across lipodystrophy subtypes.

Given the high prevalence and impact of these metabolic complications, early implementation of lifestyle interventions is essential to help mitigate disease progression and improve metabolic outcomes. Lifestyle interventions constitute the cornerstone of lipodystrophy management and should be implemented in all patients, regardless of disease subtype or access to specific therapies. Nutritional counseling focused on reducing simple carbohydrates and saturated fat intake is essential to limit postprandial hypertriglyceridemia, ectopic lipid deposition, and worsening insulin resistance [1,2]. Given the limited capacity for safe lipid storage in adipose tissue, dietary excess is preferentially diverted to the liver, muscle, and pancreas, amplifying metabolic toxicity. Regular physical activity further contributes to improved insulin sensitivity and cardiometabolic risk reduction, although exercise recommendations should be individualized according to musculoskeletal and cardiac involvement [1,2].

Multisystem involvement

Our findings further highlight the clinical relevance of extra-adipose and extra-metabolic complications. Notably, cardiomyopathy was observed in three patients: two with generalized lipodystrophy (patients with Berardinelli-Seip syndrome), both presenting with hypertrophic cardiomyopathy, and one with familial partial lipodystrophy type 3, carrying a likely pathogenic heterozygous *PPARG* variant, who developed dilated cardiomyopathy. Additionally, one patient with partial lipodystrophy and diabetes had ischemic heart disease, and one patient with Lawrence syndrome showed cardiac lipid infiltration documented by magnetic resonance imaging. This represents a relatively high prevalence of cardiac involvement, which may reflect specific genetic mutations or more aggressive phenotypes. Cardiomyopathy and other cardiovascular complications are consistent with previous reports, where major causes of mortality include cardiomyopathy, heart failure, myocardial infarction, and arrhythmias [2,20]. These findings underscore the importance of regular and comprehensive cardiac evaluation in patients with both generalized and partial lipodystrophy [2].

Beyond cardiovascular disease, autoimmune conditions were also frequent in our cohort, affecting 31.6% of patients, aligning with prior descriptions in acquired and some congenital forms of lipodystrophy [17]. This observation supports a potential immune-mediated mechanism in the pathogenesis of specific lipodystrophy subtypes. Autoantibodies directed against perilipin-1, a lipid droplet protein that regulates lipolysis, have been identified in a subset of patients with autoimmune-associated AGL; however, the precise clinical and pathogenic significance of these autoantibodies remains unclear [9,21-23]. Their consistent detection in some patients suggests they may serve as potential biomarkers of autoimmune-mediated adipocyte injury [10,23]. In our case series, two female patients with generalized lipodystrophy tested positive for anti-perilipin 1 autoantibodies. They also presented a history of multiple autoimmune conditions, including autoimmune gastritis, autoimmune thyroiditis, and autoimmune hepatitis. The coexistence of lipodystrophy and other autoimmune manifestations reinforces the hypothesis of an autoimmune basis in at least a subset of AGL patients, consistent with previous reports [10,21,22].

Acquired partial lipodystrophy (APL) has similarly been associated with autoimmune diseases such as systemic lupus erythematosus and dermatomyositis, and around 20% of patients develop membranoproliferative glomerulonephritis. While metabolic complications are less common in APL, end-stage renal disease requiring transplantation has been reported. Taken together, concomitant autoimmune disorders appear to be a major contributor to morbidity and potentially mortality in acquired forms of lipodystrophy [17].

In addition to autoimmune involvement, lipodystrophies are also associated with relevant endocrine and reproductive manifestations. Polycystic ovary syndrome (PCOS) represents a relevant endocrine manifestation in lipodystrophy, including AGL [2,17]. Severe insulin resistance and compensatory hyperinsulinemia can promote hyperandrogenism and ovarian dysfunction, resulting in PCOS-like features. Menstrual irregularities and ovarian cysts may be observed in patients with AGL, reflecting the underlying metabolic context; therefore, awareness of PCOS and related reproductive manifestations is essential for comprehensive endocrine care.

Although representing a single case in our cohort, the patient with syndromic complex lipodystrophy illustrates an important diagnostic challenge. Lipodystrophy can occur as part of multisystem genetic or neurodegenerative conditions, in which adipose tissue loss may be overshadowed by neurological, skeletal or autoimmune features [8,17]. Early recognition of the lipodystrophic phenotype in such rare syndromes is crucial, as metabolic deterioration can be disproportionately severe relative to the degree of fat loss and may progress before the underlying syndrome is fully elucidated [8,17].

Role of genetic testing and imaging

Despite recent advances, no known pathogenic variants have been identified in many patients presenting with clinical features of lipodystrophy. In our study, two patients were found to carry a confirmed pathogenic variant in *BSCL2*, one patient had a likely pathogenic variant in *PPARG*, and one patient had a VUS in *CAV1*. Twelve patients had negative genetic testing, and five were not genetically tested.

While the genetic cause can currently be identified in more than 80% of patients with CGL, in many patients with clinical features of lipodystrophy, no known mutations are detected, suggesting that either additional causative genes remain to be discovered, and/or there is a polygenic basis for the disease [10]. This difference may be explained by the fact that many cases corresponded to FPLD without a known genetic cause, the use of a limited genetic panel, and the presence of variants of uncertain significance. It is important to emphasize that a negative genetic result does not exclude a clinical diagnosis.

Of note, based on the phenotypic characterization, patients with negative genetic testing may be considered consistent with Type 1 familial partial lipodystrophy (Köbberling syndrome) [24]. This highlights that, even in the absence of known pathogenic variants, a recognizable clinical profile can support the diagnosis of FPLD1. This underscores the value of detailed phenotypic assessment in identifying Köbberling-like cases and suggests that additional genetic factors, currently unidentified, may contribute to the manifestation of this syndrome.

With regard to imaging, no firm diagnostic criteria for lipodystrophy have been established based on imaging procedures such as DXA and MRI; nevertheless, these evaluations remain valuable tools to support the diagnosis and characterize the pattern of adipose tissue loss [8]. In our cohort, 11 patients underwent DXA to quantify total body fat and regional fat distribution. As expected, patients with generalized lipodystrophy exhibited markedly reduced total and regional fat, affecting both the limbs and trunk. Among patients with suspected partial lipodystrophy, the median trunk-to-limb fat mass ratio suggested a relative preservation of truncal adipose tissue in comparison to the limbs.

Combining imaging assessments such as DXA with clinical evaluation and biochemical markers, including leptin, provides a more comprehensive approach to the identification and characterization of lipodystrophy phenotypes.

Therapeutic implications: impact of metreleptin

In this case series, the patients treated with metreleptin showed a substantial metabolic improvement, most notably in triglyceride levels, in line with findings from clinical trials [2,8]. Glycemic control also improved in all three cases, with HbA1c reductions ranging from 0.4% to 1.9%; however, the magnitude of response varied: while two patients achieved marked improvement, one patient with *BSCL2*-related CGL showed only modest glycemic benefit despite a significant fall in triglycerides. This interindividual variability suggests that metreleptin’s effects on glucose metabolism may depend on additional factors such as residual β-cell function, baseline insulin resistance, disease duration, or adherence to other therapies. Importantly, the metabolic improvements observed with metreleptin therapy should be interpreted within the context of comprehensive care. Restoration of leptin signaling reduces hyperphagia, improves insulin sensitivity, and decreases hepatic steatosis and hypertriglyceridemia; however, these effects are maximized when combined with structured nutritional and other lifestyle interventions [1].

Improvements extended beyond glucose and lipid parameters. All patients experienced reductions in hepatic enzymes, most strikingly in the patient with AGL, whose transaminases normalized from markedly elevated levels. Albuminuria also decreased consistently across the three cases, further supporting the broad metabolic and end-organ benefits of this treatment. Overall, these findings reinforce metreleptin’s role as a cornerstone therapy. Of note, the occurrence of ovarian cysts in the two female patients receiving metreleptin raises the question of whether this treatment might influence ovarian physiology. Nonetheless, causality cannot be inferred from such a small case series. Ovarian cysts have been reported as adverse events in up to 8% of female patients with generalized lipodystrophy treated with metreleptin in open-label studies and are included in the Food and Drug Administration-approved prescribing information [25]. However, the underlying pathophysiology of lipodystrophy itself may contribute to ovarian alterations. Prospective clinical studies have not shown significant changes in ovarian size or a consistent increase in cyst incidence during treatment [26]. Further studies are warranted to clarify any potential link between metreleptin therapy and ovarian cyst development.

Strengths and limitations

One of the strengths of this study is the relatively large series for a single local center (21 cases), which is a substantial number considering the rarity of the condition. In addition, the collection of detailed biochemical data, body composition assessment and comprehensive clinical history allowed for a robust characterization of the cohort. However, the study also has several limitations. First, its retrospective design and small sample size limit the generalizability of the findings. The single-center design and referral-based nature of this cohort also introduce a potential selection bias, as patients followed in a tertiary endocrinology clinic may represent more severe or complex cases. Follow-up was not uniform across patients, and conclusive genetic testing was lacking in the majority of cases. Moreover, data on leptin levels, liver imaging and body composition assessment through DXA were incomplete, which restricted a more comprehensive assessment of metabolic and organ-specific complications. Nonetheless, it reflects the real-world challenges in diagnosing and managing lipodystrophy and supports the need for multicenter collaborations and improved genetic screening strategies. A further limitation of this study is the lack of a systematic assessment of quality of life, functional status, and patient-reported outcomes. Although metabolic and imaging indices provide valuable information on disease burden, they do not fully capture the impact of lipodystrophy on patients’ daily living. This represents an important area for future research.

Of note, although sex was recorded and the cohort was predominantly female, no sex- or gender-specific comparative analyses were performed due to the small sample size.

## Conclusions

This case series highlights that lipodystrophies are often underdiagnosed and that many patients already present with complications at the time of diagnosis. It emphasizes the importance of educating clinicians to recognize characteristic phenotypic patterns, ensuring access to genetic testing, implementing systematic screening for metabolic and cardiovascular complications, and improving the availability of specific therapies such as metreleptin.

Taken together with other European registries, our findings suggest broadly consistent patterns in the epidemiology and metabolic burden of lipodystrophy across European populations, although minor differences may reflect referral practices or case severity.
